# Shark tooth regeneration reveals common stem cell characters in both human rested lamina and ameloblastoma

**DOI:** 10.1038/s41598-019-52406-z

**Published:** 2019-11-04

**Authors:** Gareth J. Fraser, Samar S. Hamed, Kyle J. Martin, Keith D. Hunter

**Affiliations:** 1 Department of Animal and Plant Sciences, University of Sheffield, Sheffield, UK; 2 Integrated Biosciences, School of Clinical Dentistry, University of Sheffield, Sheffield, UK; 3 Oral Biology and Pathology, University of Pretoria, Pretoria, South Africa; 4 Department of Biology, University of Florida, Gainesville, FL USA

**Keywords:** Cell proliferation, Stem cells

## Abstract

The human dentition is a typical diphyodont mammalian system with tooth replacement of most positions. However, after dental replacement and sequential molar development, the dental lamina undergoes apoptosis and fragments, leaving scattered epithelial units (dental lamina rests; DLRs). DLRs in adult humans are considered inactive epithelia, thought to possess limited capacity for further regeneration. However, we show that these tissues contain a small proportion of proliferating cells (assessed by both Ki67 and PCNA) but also express a number of common dental stem cell markers (Sox2, Bmi1, β-catenin and PH3) similar to that observed in many vertebrates that actively, and continuously regenerate their dentition. We compared these human tissues with the dental lamina of sharks that regenerate their dentition throughout life, providing evidence that human tissues have the capacity for further and undocumented regeneration. We also assessed cases of human ameloblastoma to characterise further the proliferative signature of dental lamina rests. Ameloblastomas are assumed to derive from aberrant lamina rests that undergo changes, which are not well understood, to form a benign tumour. We suggest that dental lamina rests can offer a potential source of important dental stem cells for future dental regenerative therapy. The combined developmental genetic data from the shark dental lamina and ameloblastoma may lead to the development of novel methods to utilise these rested populations of adult lamina stem cells for controlled tooth replacement in humans.

## Introduction

The potential for tooth regeneration in vertebrates is both widespread and incredibly diverse. The capacity for multiple tooth regeneration is reduced in higher positions within the vertebrate phylogeny, i.e. mammals have a limited system typically of just a single round of tooth replacement^1^. Fishes generally have a greater capacity for tooth regeneration and most have continuous regeneration throughout life - an unlimited supply (polyphyodonty)^2,3^. It is thought that the necessity for occlusion in diet-specialised mammals led to the restriction of multiple replacement generations, although some mammals still retain the capacity for further tooth production e.g. elephant, manatee, rock wallaby^1,4^, others have reduced the dentition to a single generation of teeth (monophyodonty), e.g. rodents^1^. A general trend is therefore observed in vertebrates that from fishes to amniotes there is an overall reduction in the number of tooth generations^1,4^. The production of teeth and the regenerative capacity of the dentition arises from a specialized epithelial structure, formed early in the development of vertebrate mouth, called the dental lamina^4^. The dental lamina emerges from the initial thickened oral epithelia from which early tooth buds emerge, a process common to all toothed vertebrates^4^. For animals with continuous tooth production, the dental lamina is persistent or at least has the ability to regrow to accommodate the appearance of new teeth^2–7^. However, in animals with a limited tooth supply, i.e. monophyodont and diphyodont species, the dental lamina is non-persistent and degrades over time^5,6^.

**Figure 1:**
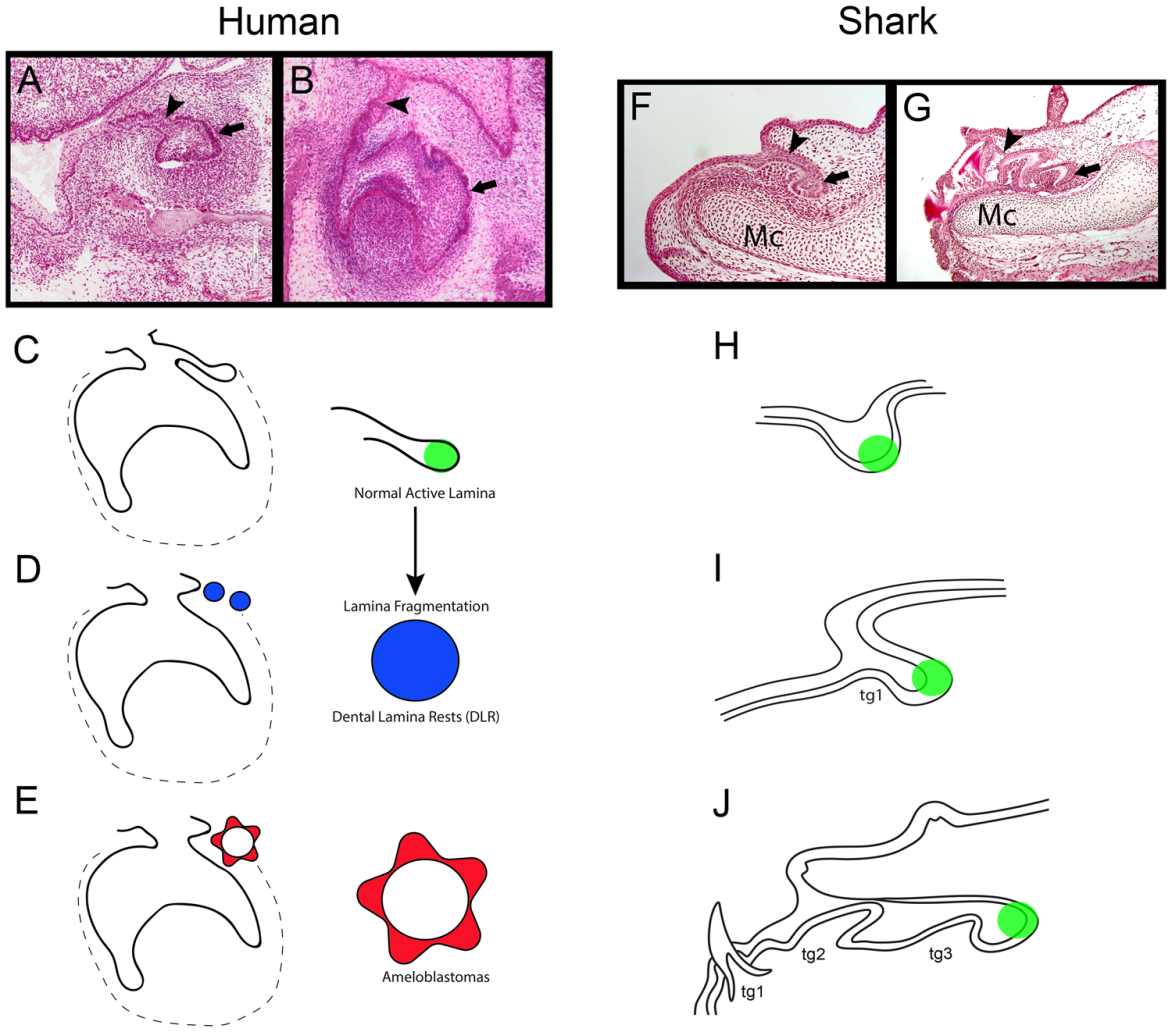
Varied fates of the dental lamina in Human (**A–E**) and Shark (**F**–**J**). Representative photomicrographs (H&E stained sections) of human tooth development. (**A,B**) Enamel organ epithelium (Arrow in **A,B**) and the dental lamina (Arrowhead in **A,B**) fragments after tooth development to become rested lamina (**D**). Normal active lamina in human is represented by the successional lamina in C (green). Shark tooth development (**F,G**; sagittal section, lower jaw) progresses with continued growth and proliferation of the dental lamina from the first tooth stage (**F**; Arrowhead) and tooth regeneration initiates at the site of active lamina (Arrow in F and G, and green colour in **H**–**J**). Active and normal lamina in both human and shark houses a progenitor niche (green), retained throughout life in the shark for continuous tooth development (see **G**). In humans, the dental lamina fragments after tooth development of the first or second-generation tooth set into dental lamina rests (DLRs; blue). DLRs can further develop into tumorigenic ameloblastoma (Red star). tg = tooth generation. Mc = Meckel’s cartilage.

We focus here on the comparison of two systems (i) the limited human dentition and (ii) the actively regenerative shark dentition^2,3^ to understand the stem cell link between the shared epithelial dental lamina and the derivatives of these divergent dental systems (Fig. 1). Humans have regenerative capacity in the early forming dental lamina^5^ but the current consensus is that replacement potential is lost after the second generation (incisors, canines, premolars) and after formation of the single generation of molars in sequence (M1-3)^6^. The dental lamina in humans undergoes apoptotic disintegration and breaks down to prevent further *de novo* regeneration after the development of the partial second generation^5^. Therefore, we address whether the remnants of fragmented human dental lamina - called dental lamina rests (DLRs: those restricted epithelial cell populations) - are commonly present and whether they can retain a level of regenerative potential and progenitor activity that could be utilised by future dental therapies. These DLRs are commonly found within the dental follicle (DF) of unerupted teeth and in the connective tissues which comprise the wall of the dentigerous cyst (DC) of the jaws.

It is known that these rested cell populations possess some ability to further proliferate as they can form a number of aberrant structures in the human oral cavity, including odontomas and ameloblastomas^8^; these odontogenic tumours are considered hamartoma or benign neoplasms respectively, but can be very destructive^9^. We aimed to compare these epithelial remnants (DLRs) with epithelia associated with both human ameloblastoma, and a continuously active dental lamina present in the shark (*Scyliorhinus canicula*) necessary for lifelong tooth regeneration^2,3^. This comparison is significant to recognise the common stem-like factors within these tissues that may indicate a retained capacity for regeneration in adult human DLRs. Recent data suggests that the shark dental lamina actively and rapidly produces teeth in a conveyor belt-like process that (i) is governed by a highly conserved set of core genes shared from sharks to mammals^3^ and (ii) fed by populations of progenitor cells for continuous production^2^. We hypothesise that human DLRs, common in adult oral tissues, are a potential source of stem cells (progenitors) fated for dental differentiation and could be utilised for novel tooth regeneration in humans after tooth loss.

## Results

**Figure 2:**
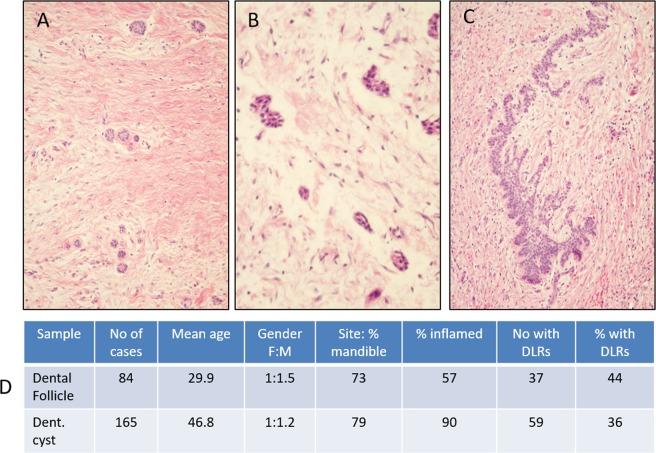
(**A–C**) Photomicrographs of H&E stained Dental follicles containing dental lamina rests (Overall magnification: A x200, B x400, C x400), with panel A and C demonstrating the variability in size and extent in DLRs seen. (**D**) Summary of the clinical demographics of the Dental Follicle and Dentigerous Cyst cases, with prevalence of DLRS from all cases accessions from 2010 to 2014.

In order to explore the regenerative potential of DLRs, we assessed a cohort of dental follicles (DF) and dentigerous cysts (DC) for DLR content. We identified 84 dental follicle cases from 2004–2014 and 165 dentigerous cyst cases from 2010–2014 for assessment. Summary demographic details of these cohorts are presented in Fig. 2. We selected a subset of the dental follicles containing DLRs for further analysis of the expression of a number of proliferation and stem cell markers which have been identified in the shark dental lamina. Furthermore, we compared the proliferative and stem characters of DLRs to human ameloblastoma, a known derivative of aberrant DLR proliferation^8^.

### Extent of DLRs in adult human tissues

Assessment of the whole cohort of dental follicles (DFs) and dentigerous cysts (DCs) showed that 44% and 36%, respectively, contained DLRs of varying size and to varying extents (Fig. 2). Most cases, in both groups (73% and 79%), were in the mandible and a majority showed some extent of inflammation. The proportion of dental follicles containing DLRs declined with age: 34/45 (76%) of cases from patients under 25 years of age contained DLRs, whilst only 14/38 (37%) of those 25 years or older did so. Cases with inflammation were excluded for the further analysis as we wished to only study rests that were not under an inflammatory stimulus, which can act as a profound stimulus of proliferation. The details of the final selected cohort of five cases are shown in Table 1.

**Table 1:** The final patient cohort in the study. DLR = Dental Lamina Rest, A = Ameloblastoma.

Case	Diagnosis	Site	Age	Gender	Comment
DLR1	Dental Follicle	Mandibular 3^rd^ molar	25	M	—
DLR2	Dental Follicle	Maxillary Canine	5	M	—
DLR3	Dental Follicle	Mandibular 3^rd^ molar	15	M	—
DLR4	Dental Follicle	Mandibular premolar	16	M	—
DLR5	Dental Follicle	Maxillary primary molar	5	M	—
A1	Ameloblastoma	Post maxilla	49	M	Conventional type, follicular pattern
A2	Ameloblastoma	Post mandible	54	M	Conventional type, follicular pattern
A3	Ameloblastoma	Post mandible	48	M	Conventional type, follicular pattern
A4	Ameloblastoma	Post mandible	43	M	Conventional type, follicular pattern
A5	Ameloblastoma	Mandible premolar	62	M	Conventional type, follicular pattern

### Proliferation in DLRs and Ameloblastoma

**Figure 3:**
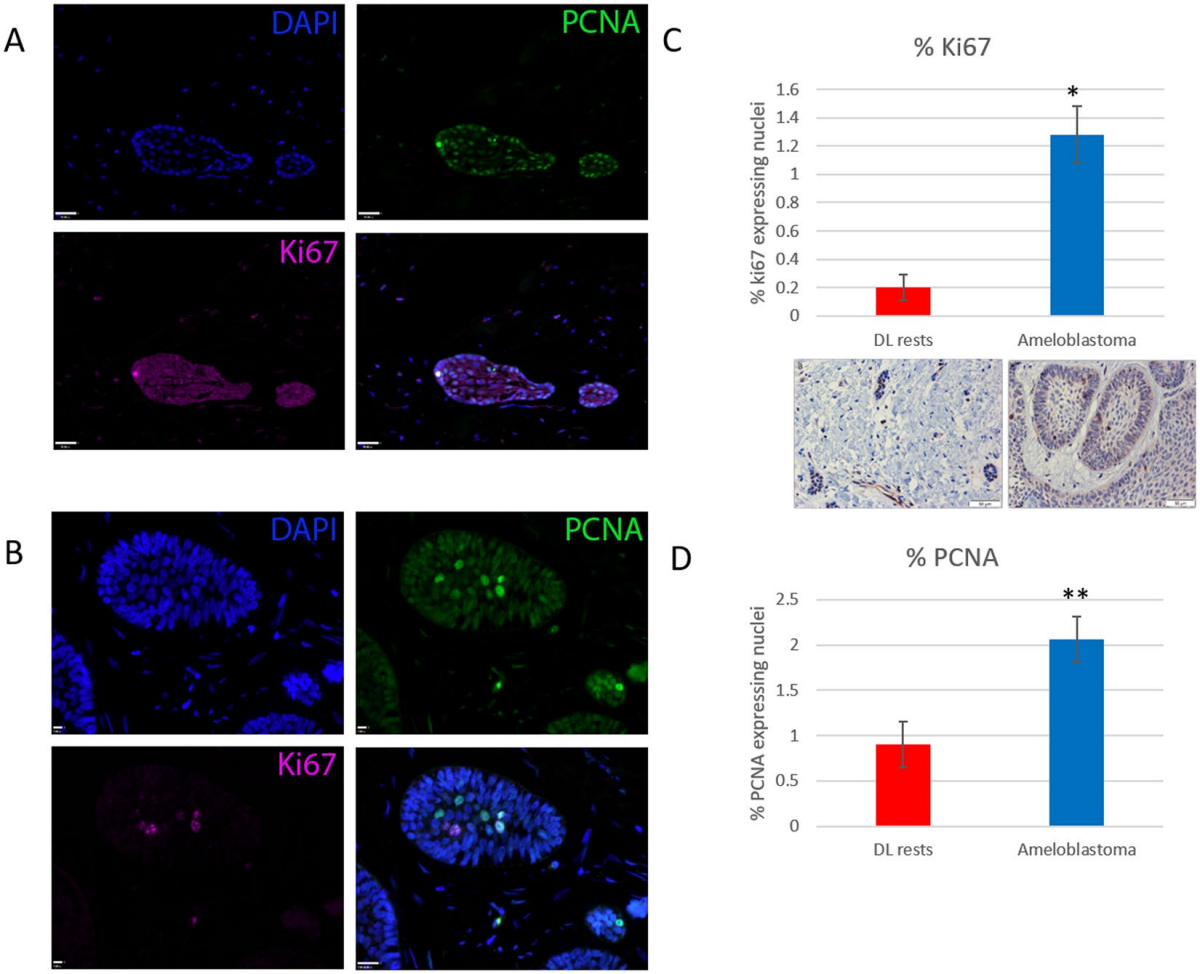
Ki67 and PCNA expression in dental lamina rests (**A**) and ameloblastoma (**B**). The percentage of nuclei expressing each marker in DLRs and Ameloblastoma is shown in panel C (Ki67, with matched immunohistochemistry) and panel D (PCNA). *p < 0.001 and **p < 0.05, respectively.

The proliferative potential of the DLRs and Ameloblastoma was assessed by expression of Ki67 and PCNA. Only cells expressing a high level of either marker were assessed as positive. For both Ameloblastoma and DLRs cell counts were performed on a representative set of ‘follicles’, with approximately 300–500 cells across at least 5 positive fields. Both markers demonstrated a significantly higher percentage of proliferating cells in ameloblastoma when compared with DLRs. The proportion (%) of PCNA positive cells was higher than for Ki67 in both cohorts (Fig. 3). In DLRs, the proliferating cells were present on the periphery of the nests, however, in ameloblastoma, proliferating cells could be found in either the peripheral cells or intermediate stellate reticulum-like areas.

### Expression of dental stem markers in DLRs and ameloblastoma

**Figure 4:**
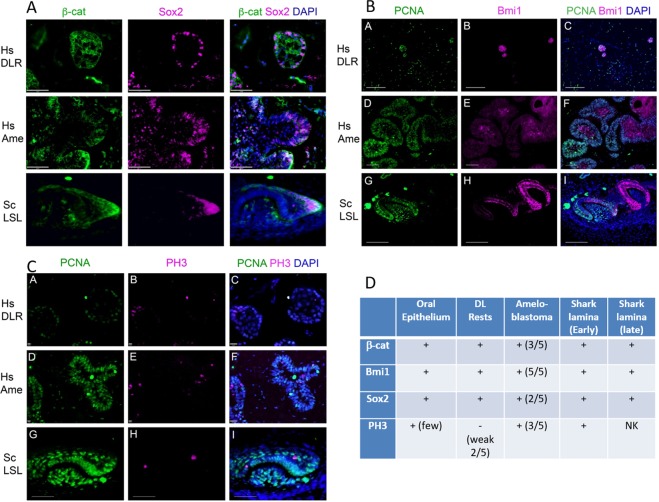
Photomicrographs of immunofluorescent assessment of PCNA, SOX2, BMI1, β-Catenin and PH3 expression in dental lamina rests (DLR), ameloblastoma (Ame) and shark dental lamina (LSL; lower successional lamina), with DAPI as a nuclear counterstain. (Panels A–C). Panel D presents a summary of expression of all markers in the various groups. NK = not known. Hs=Human, (*Homo sapiens*); Sc=Shark, (*Scyliorhinus canicula*).

We investigated whether a number of stem cell-associated markers were expressed in DLRs, and ameloblastoma compared to shark (*S. canicula*) dental lamina (Fig. 4). We assessed the expression of SOX2, PH3, β-catenin, and BMI1 to appreciate the comparative genetic profiles of these disparate dental cell populations. Expression in the overlying oral epithelium was included where this was present for assessment (3/5 DLR cases), as a control (data not shown). The spatial pattern of expression varied, dependent on the marker used; SOX2 was expressed in the nuclei of peripheral cells; β-catenin was expressed in the cytoplasm of both peripheral and central cells (Fig. 4A); BMI1 was predominantly expressed in the cells in the centre of epithelial nests (Fig. 4B), whilst PH3 was expressed in the nucleus of occasional peripheral and central cells (Fig. 4C).

### Expression of dental stem markers in active shark dental lamina

To appreciate the shifts in proliferative and progenitor cell characteristics in both active and rested dental epithelium, we compared the immunofluorescence signature of stem markers in human rested lamina (DLRs) with the continuously active dental lamina in the polyphyodont shark (catshark; *Scyliorhinus canicula*). This rapidly productive multi-generational dental conveyor-belt requires a continuously active dental lamina to maintain both progenitor cell populations and cyclical turnover of perpetual tooth development. The shark dental lamina is a continuous and permanent structure that covers the pre-made teeth prior to functionality and additionally contains epithelial progenitor cells associated with the successional lamina where new teeth are initiated (Fig. 1). We observed a very similar pattern of immuno-localisation of PCNA, Sox2, Bmi1, and PH3, in the shark dental lamina (Fig. 4), suggesting that these genetic signatures are common to highly regenerative cell populations that contribute to the dental lamina. The shark dental lamina can be thought of as a set of developmental compartments, with the free-end of the lamina sheet (termed the successional lamina) responsible for the induction and continued activation of new tooth replacements. Sox2 is localised to peripheral successional lamina cells (equivalent to an outer dental epithelium), and these cells are thought to be a sub-set of progenitor cells that transition to the successional stem niche located within this region (Fig. 4A^2^;).

Sox2 and cytoplasmic (activated) β-catenin (Ctnnb1) are regionally co-expressed in the hatchling catshark (Stage 34^2^) successional lamina (SL) at the site of new tooth turnover. The expression pattern of sox2/β-catenin in the deep SL is consistent with the location of the shark dental stem cell niche^2^. β-catenin expression is diffuse throughout the lingual end of the SL (Fig. 4A), marking the site at which new teeth are initiated. Sox2 expression is more restricted in this region, present in the outer dental epithelial cells (peripheral cells) of the lingual extent of the SL, where the dental stem niche governs the production of new teeth. Intriguingly, the expression of sox2/β-catenin in the shark SL is consistent with the proliferative, columnar peripheral edges of the ameloblastomas (Fig. 4A), whereas the DLRs show more peripherally restricted and limited sox2+ cells compared to the activated β-catenin expression present in the cytoplasm of entire DLRs. A number of studies have demonstrated expression of SOX2 in ameloblastoma. This was initially identified in a transcriptomic screen, which also demonstrated expression of a number of other early dental epithelial markers^10^.

Bmi1 expression in the shark dental lamina is restricted to the inner dental epithelium (IDE) of developing teeth and weakly expressed in the cells that overlap the region of the SL stem cell niche, however little or no expression is observed in the highly proliferative (PCNA+) middle dental epithelial (MDE) cells of the SL. In contrast, Bmi1 is confined to the core of the ameloblastoma nests (Fig. 4B) and not highly expressed in the proliferative periphery of the structures.

Similar to that observed in both the ameloblastoma and DLRs, the intermediate cells (or middle dental epithelium, equivalent to the central cells in Ame/DLRs) of the shark successional lamina show a similar pattern of Bmi1, PH3 and PCNA expression (Fig. 2), which indicates that this is a dynamic and highly proliferative region of the lamina, capable of maintaining stem cells necessary for continued tooth production. The dynamic dental lamina in the shark is a highly proliferative unit, which accounts for both the continuous growth of the lamina itself and the continuous production of teeth (Fig. 1). Therefore, a comparative expression profile in human tissues (DLRs and ameloblastomas) may be linked to a retained capacity for further proliferation and developmental or regenerative potential in rested human cell populations.

## Discussion

### Dental lamina rests as a potential source of epithelial stem cells

The assessment of the pericoronal tissues either from dental follicles or associated with dentigerous cysts has demonstrated the extent of the presence of DLRs in human tissues. These have, along with other potential sources of dental epithelial stem cells (e.g. epithelial cell rests of Mallasez^11^), been suggested as a potential source of stem cells for regenerative applications, albeit that there are significant issues in the availability and accessibility of these cells. Whilst their existence has been long accepted, some suggest that whilst DLRs hold potential for dental regeneration, that their persistence is in doubt^8,9^. The extent of DLRs in the tissues, which we have described, indicates that this may be less of a problem as a significant proportion of the tissues did contain DLRs. Indeed, given that they are often very focal, and that for most of the cases, only one diagnostic slide was assessed, this may be an underestimate of prevalence of DLRs. Although, a different population of epithelial cells to the lamina rests, studies have suggested that epithelial rests of Malassez, associated with the periodontal ligament, decline with age^11^. Our observation that the proportion of DFs with DLRs reduced with age is potentially significant and may be a limiting factor: yet over one third of cases from patients older than 25 contained DLRs, demonstrating that these cell rests do persist after the completion of tooth formation.

The extent of proliferation in DLRs is very poorly described in the literature. We have found that Ki67 and PCNA-expressing cells are present in the DLRs, but they are significantly fewer than in ameloblastoma (Fig. 3). It is generally accepted that the proliferation fraction in ameloblastoma is relatively low^12^. The reported proportion of proliferating cells in our cases is similar to that in the literature^13–15^. However, in some reports, there are wide variations, which seem to depend on combinations of the antibody clones used and the cut-offs in the assessment of staining^16^. We also found that the cells, which express these markers, were peripheral cells, a feature also generally accepted in the literature, although some exceptions have been reported^17^.

In general, those who have assessed Ki-67 and PCNA expression have found that a higher proportion of cells express PCNA, in some cases markedly higher^18,19^. These differences are due to variation in the phases of the cell cycle which these markers are expressed: Ki-67 is expressed in proliferating cells and is preferentially expressed during late G1, S, G2 and M phase. Cells in G0 lack Ki-67 expression. The expression of PCNA is more constrained, particularly seen in G1/S, but PCNA also performs a number of other functions e.g. cell cycle-dependent function and DNA replication, which may explain the higher proportion of expressing cells. In our tissues, the proportion of high PCNA expressing cells was very low (as quantified in Fig. 3), but many other cells express PCNA at a low level, (see Fig. 4, panels B and C) in a similar proportion to that seen in the shark dental lamina. The significance of this is not known.

### The shark dental lamina as a model for active and highly productive tooth regeneration

The shark is an exciting emerging model for studies of tooth development and regeneration due to their incredibly rapid production of teeth, and the perpetual nature of their *de novo* tooth regenerative capabilities^2–4^. The recent genomic advances to members of the elasmobranch lineage^20,21^ have begun to deepen our knowledge of these organisms, thus paving the way for further progress in comparative developmental and genomic biology. Thus, the comparative power of these developmental models to inform the evolution and development of mammalian systems will allow translational innovations to develop directly from our knowledge of these unusual model organisms.

Dental lamina-derived cell populations from sharks to humans have seemingly different states of proliferation and longer-term function, in fact the expression of core stem markers suggests that these cell populations may have much more in common. A shared genetic signature could underlie the regenerative and stem potential in these seemingly rested populations of human lamina, an exciting prospect for future dental therapies. Further work on the regulation of tooth regeneration in sharks will ultimately reveal additional markers and interactions that may or may not be present in the rested human cell populations. One exciting aspect of this research is the comparative genetic conservation of these seemingly disparate vertebrate tissues (shark and human dental lamina), and any shifts in the signalling that relate to the capacity for perpetual tooth regeneration, e.g. in sharks, may offer new target markers to study further in a mammalian model, or more specifically in humans, to fully appreciate the mechanism of lost regenerative potential for translational developments.

## Methods

### Clinical cohort

All cases of dental follicle accessioned from 2004 to 14, and dentigerous cyst accessioned from 2010 to 2014 and five cases of ameloblastoma were retrieved from the Diagnostic Archive in the Oral Pathology Diagnostic Service, Charles Clifford Dental Hospital, Sheffield. Ethics approval for the use of biopsy tissues in this study was obtained from The West Glasgow LREC (ref: 08/S0709/70). The Ethics Approval waived the need for specific consent as the material was fully anonymized and surplus to diagnostic requirements. Demographic data was collected including age, gender, site, tooth association, and the DF and DC sections were examined for the presence of inflammation and dental lamina rests, which were assessed by number, size and distribution. From this cohort, five uninflamed DF cases were selected for further analysis, on the basis of the number of DLRs available for assessment (Table 1). In three cases, overlying oral epithelium was also present, which acted as an internal control.

### Shark husbandry

The University of Sheffield is a licensed establishment under the Animals (Scientific Procedures) Act 1986. All animals were culled by approved methods cited under Schedule 1 to the Act and approved by the University of Sheffield. Small-spotted catshark (*Scyliorhinus canicula*) embryos were obtained from Station Biologique de Roscoff, Roscoff, France. Embryos were raised and staged^22^ in an artificial saltwater aquarium at 12 °C at the University of Sheffield, Sheffield, United Kingdom. Embryos were killed with MS-222 (tricaine) at 300 mg/L and fixed in 4% (wt/vol) paraformaldehyde overnight at 4 °C.

### Immunofluorescence

Dewaxed 5 µm paraffin slides were subjected to heat-mediated antigen retrieval in hot 0.01 M sodium citrate pH = 6.0 for 20 min before blocking and subsequent antibody labelling. Primary antibody labelling with rabbit anti-Sox2 (Abcam ab97959) 1:500, rabbit anti-Ki67 (santa Criz 15402) 1:200, rabbit anti-Bmi1(Abcam ab127934) 1:500, mouse anti-PCNA (Abcam ab29) 1:2,000, or mouse anti-active β-catenin (ABC; Merck 05–665) 1:500 was carried out overnight at 4 °C. Secondary antibody incubation with goat anti-rabbit AlexaFluor-647 (Thermo A-21245) 1:250 or goat anti-mouse AlexaFluor-488 (Thermo A-11-001) 1:250 was carried out for 1 h at room temperature. Slides were counterstained with 1 μg/mL DAPI (Sigma D9542) and mounted in Fluoromount (Sigma F4680). Imaging was carried out on an Olympus BX51 upright epifluorescent microscope and visualized with the software Volocity 6.3.
